# TRF1 and TRF2 binding to telomeres is modulated by nucleosomal organization

**DOI:** 10.1093/nar/gkv507

**Published:** 2015-05-20

**Authors:** Alessandra Galati, Emanuela Micheli, Claudia Alicata, Tiziano Ingegnere, Alessandro Cicconi, Miriam Caroline Pusch, Marie-Josèphe Giraud-Panis, Eric Gilson, Stefano Cacchione

**Affiliations:** 1 Department of Biology and Biotechnology ‘Charles Darwin’, Sapienza University of Rome, 00185 Rome, Italy; 2 Institute Pasteur-Fondazione Cenci-Bolognetti, Sapienza University of Rome, 00185 Rome, Italy; 3 Adolf-Butenandt-Institut, Ludwig-Maximilians-Universität, 80336 München, Germany; 4 Institute for Research on Cancer and Aging, Nice (IRCAN) CNRS UMR 7284/INSERM U1081, University of Nice Sophia Antipolis, 06107 Nice, France; 5 Department of Medical Genetics, Hospital, CHU of Nice, 06202 Nice, France

## Abstract

The ends of eukaryotic chromosomes need to be protected from the activation of a DNA damage response that leads the cell to replicative senescence or apoptosis. In mammals, protection is accomplished by a six-factor complex named shelterin, which organizes the terminal TTAGGG repeats in a still ill-defined structure, the telomere. The stable interaction of shelterin with telomeres mainly depends on the binding of two of its components, TRF1 and TRF2, to double-stranded telomeric repeats. Tethering of TRF proteins to telomeres occurs in a chromatin environment characterized by a very compact nucleosomal organization. In this work we show that binding of TRF1 and TRF2 to telomeric sequences is modulated by the histone octamer. By means of *in vitro* models, we found that TRF2 binding is strongly hampered by the presence of telomeric nucleosomes, whereas TRF1 binds efficiently to telomeric DNA in a nucleosomal context and is able to remodel telomeric nucleosomal arrays. Our results indicate that the different behavior of TRF proteins partly depends on the interaction with histone tails of their divergent N-terminal domains. We propose that the interplay between the histone octamer and TRF proteins plays a role in the steps leading to telomere deprotection.

## INTRODUCTION

Telomeres represent the solution to the problems encountered by eukaryotic cells switching from circular to linear chromosomes (1). Due to the need of an RNA primer to start copying DNA, DNA polymerases are unable to entirely replicate eukaryotic genome; in addition, chromosome ends need to form specific protective structures to avoid inappropriate processing by DNA repair enzymes. In most eukaryotes, telomeres consist of short G-rich sequences repeated in tandem, ending in a single-stranded protrusion named 3′ G-overhang; the solution to the telomere erosion problem is provided by the enzyme telomerase, a reverse transcriptase which adds telomeric repeats onto the 3′ ends of chromosomes (2). The formation of a protected structure requires the binding of specific proteins; in mammals, capping is assured by the protein complex shelterin, composed of the six proteins TRF1, TRF2, Rap1, POT1, TIN2 and TPP1 (3).

Human telomeres at birth are 10–15 kb long, consisting of 5′-TTAGGG-3′ double-stranded repeats ending in 50–500-nt long 3′ G-overhangs (4). In somatic cells telomerase is inactive and telomeres shorten at every replication round, until they reach a critical length that triggers a DNA damage response (DDR) pathway leading to the permanent cell cycle arrest known as replicative senescence (5,6). If checkpoints failed, cells bypass senescence and continue dividing. The ongoing end-erosion results in chromosome fusions and high genomic instability, a state known as crisis, characterized by massive cell death. Rarely, pre-malignant cells escape crisis by acquiring a mechanism to maintain telomeres, in most cases by reactivating telomerase (7). Telomere maintenance confers unrestrained proliferation capacity, one of the main hallmarks that characterize cancer cells (8).

Protection from DDR activation and instability is assured by the proteins that compose shelterin: TRF1 and TRF2 that bind as homodimers TTAGGG double-stranded repeats, the POT1/TPP1 heterodimer that binds single-stranded G-overhangs, Rap1 that binds TRF2 in a 1:1 ratio and TIN2 that connects together the shelterin components (3). The main role in shelterin binding to telomeres is played by TRF1 and TRF2 (9,10). They share the same architecture, with a Myb-SANT DNA binding domain, a hinge domain and a dimerization domain. The two proteins diverge in their N-terminal domain, rich in acidic residues in the case of TRF1, rich in Gly and Arg residues (the GAR domain) for TRF2. Although the Myb-SANT DNA binding domains of TRF1 and TRF2 recognize TTAGGG repeats in a nearly identical way (11), there are several differences in the way the two proteins bind telomeric DNA. Despite the similarities, the affinity of TRF1-Myb for telomeric DNA is almost 4-fold higher than TRF2-Myb (12). TRF2 induces and recognizes positive DNA supercoiling (13,14), a property inhibited in TRF1 by its N-terminal acidic domain (15), as well as Holliday junctions (16). Moreover, TRF2 has been proposed to stabilize the t-loop, a lariat structure that sequesters the G-overhang by invasion of the upstream TTAGGG double-stranded region (17,18). Due probably to the flexible hinge domain, the two Myb-SANT domains of a TRF1 dimer can bind distant half-sites independently from orientation, even on different DNA molecules (19,20). The binding to human telomeres of shelterin proteins protects from several DDR and repair pathways (21). The presence of TRF2 at telomeres is necessary to suppress the ataxia telangiectasia mutated (ATM)-dependent DDR pathway (22) and the non-homologous end joining (NHEJ) DNA repair pathway (23). TRF2 depletion results in the activation of the ATM kinase pathway and in the recruitment at telomeres of DDR factors such as γ-H2AX and MDC1; the consequent phosphorylation cascade leads to the activation of P53 and to replicative senescence. If the P53 pathway is mutated, telomere deprotection results in chromosome fusions by NHEJ. Different TRF2 domains mediate the inhibition of these two pathways. ATM activation is repressed by the dimerization domain TRFH, whereas NHEJ is inhibited by the C-terminal region of the hinge domain, iDDR (24).

The current view is that telomeres dynamically interchange between different structural states as a function of telomere length and cell cycle progression (25–27). Long telomeres adopt a closed structure that protects chromosome ends from DDR and NHEJ. The exact nature of this structure remains undefined, although it might coincide with the t-loop (18,28). Deprotection of a single telomere is a stochastic event (29) that activates a peculiar DDR signaling that does not contribute to G2/M checkpoint (30) and whose frequency increases with telomere shortening (31). The intermediate deprotected state is characterized by DDR activation without telomere fusion, a situation that can be explained as a partial loss of function of TRF2; accumulation of DDR signal at five telomeres triggers the P53 pathway leading to replicative senescence (31). A further loss of function of shelterin determines the fully deprotected state of telomeres leading to telomere fusions.

At present, the organization of human telomeric chromatin in protected and deprotected states is mostly unknown. Nucleosomes are present along the telomere in a peculiar tight organization (32–34). Nucleosome spacing at telomeres is ∼160 bp, thus ∼20–40 bp shorter than in bulk chromatin. The characteristics of telomeric nucleosomes and their interplay with telomeric proteins have been extensively studied *in vitro*. Telomeric nucleosomes are less stable than bulk nucleosomes (35–38), occupy multiple isoenergetic positions (37,39,40) and are intrinsically mobile (41). In addition, yeast and human telomeric repeats disfavor nucleosome formation in yeast minichromosomes (42). The yeast telomeric protein Rap1 (43) and the human telomeric protein TRF1 (44) are able to recognize telomeric repeats on the nucleosome. TRF1 is also able to induce the sliding of nucleosomes to adjacent sites (45), whereas TRF2 increases the spacing between nucleosomes when assembled *in vitro* (34) and when overexpressed *in vivo* (34,46).

To evaluate the role played by nucleosomes at telomeres, it must also be taken into consideration that several post-translational histone modifications have been associated with telomere shortening and the establishment of DDR at telomeres. In mouse cells telomere shortening correlates with a significant decrease of two heterochromatic marks at telomeres and subtelomeres, H3K9me3 and H4K20me3, and with the increase of H3 and H4 acetylation (47). Knockout of human Sirt6, a Nad^+^-dependent histone deacetylase, leads to hyperacetylation of H3K9 and H3K56, and results in telomere fusions and premature senescence (48). Although the epigenetic state of human telomeres has yet to be fully elucidated (27), these data suggest that the presence of histone marks generally associated with a heterochromatic state, such as histone hypoacetylation, is essential for the protective capping of human telomeres. Telomere deprotection, as a consequence of shelterin loss or of telomere erosion, leads to the activation of ATM and/or ATR signaling pathways and to the phosphorylation of the histone variant H2AX (γ-H2AX). This is one of the most evident modifications associated with DDR along the genome, not only at telomeres (49). The enzymatic cascade following DNA damage signaling leads to other post-translational histone modifications, such as H2A and H2AX ubiquitination (50,51).

How this dynamically changing telomeric chromatin environment affects shelterin binding and therefore the regulated transition from a protected to a deprotected telomere state is a relevant issue in telomere biology.

Here we address the question of whether telomeric nucleosomes affect the binding of TRF1 and TRF2 to telomeric DNA, by means of *in vitro* models of nucleosome assembly. We found that the presence of nucleosomes interferes not only with the binding of TRF proteins on the DNA wrapped around the histone octamer but also on the recognition of linker DNA. We also show that the binding behavior in a chromatin environment is different between TRF1 and TRF2 and that this depends on the divergent N-terminal domains of the two proteins and on the histone tails. Finally, using an *in vitro* assembly system, we show that TRF1 is able to remodel telomeric chromatin by altering nucleosome spacing.

## MATERIALS AND METHODS

### DNA fragments and nucleosome reconstitution

The Tel8-L DNA fragment was prepared as described (36,44). To obtain the Tel2-601-Tel2 construct the plasmid pUC18/601-201 was amplified by polymerase chain reaction (PCR) using the primers E-601L (5′-TCGAATTCTTAGGGTTAGGGTTACCCTGGAGAATCCCGGT-3′) and B-601R (5′CTGGATCCTAACCCTAACCCTAAGCACAGGATGTATATATCTGA-3′). The 3′ part of the primers was complementary to the ends of the 601 sequence, whereas the 5′ end contained two and a half copies of the telomeric sequence TTAGGG and the EcoRI (E-601L) and BamHI (B-601R) sites, respectively. The PCR product was digested with EcoRI and BamHI and cloned in pUC18.

The DNA fragments were extracted from the plasmid by cutting with EcoRI and BamHI, labeled by filling in the ends with Klenow enzyme and [α-^32^P]dATP, gel-purified and reconstituted into nucleosomes (43). Briefly, 1 μg of H1-free nucleosomal particles, prepared from chicken erythrocytes according to previously described protocols (52), was mixed with 0.1 picomoles of labeled DNA probe, in 1-M NaCl, 20-mM Hepes pH 7.9, 0.1% Nonidet-P40, 100-μg/ml bovine serum albumin (BSA), in a final volume of 10 μl. After incubation at room temperature for 30′, the salt concentration was lowered to 0.1-M NaCl by sequential additions every 10′ of 20-mM Hepes pH 7.9, 0.1% Nonidet-P40 (2, 4, 8, 16, 30, 30 μl).

### Trypsin digestion of the histone tails

Histone tails were digested as described by Yang *et al*. (53), using trypsin attached to agarose beads (TAB Sigma). Forty microliter of nucleosomes prepared from chicken erythrocytes (1 μg/μl) was incubated with TABs for 30 min at room temperature and then briefly microcentrifuged (12 000 rpm for 2 min) to remove the beads. Removal of core histone tail domains was verified by sodium dodecyl sulfate–18% polyacrylamide gel electrophoresis (SDS–18% PAGE) and Coomassie staining (Supplementary Figure S1).

### Protein purification

6xHis-tagged TRF1, TRF2, TRF1-Myb (371–439), TRF2-Myb (441–500), TRF2^AΔB^ (TRF1 2–67 TRF2 47–500) and TRF2^ΔB^ (47–500) were expressed in BL21(D3) cells and purified as described (11,13). After several washes with Wash Buffer (50-mM Hepes [pH8], 10-mM β-mercaptoethanol, 500-mM KCl, 20-mM Imidazole, 1-mM phenylmethylsulfonyl fluoride (PMSF), 10% Glycerol), the protein was eluted with the same buffer containing 300-mM Imidazole and dialyzed against Wash Buffer. Protein concentration was assessed by Bradford assay (SIGMA). The purity of the isolated proteins has been verified by SDS-PAGE electrophoresis followed by Coomassie staining (Supplementary Figure S2).

### Electrophoretic mobility-shift assay

Binding reactions were carried out by incubating the indicated amounts of protein and reconstituted nucleosome or tailless nucleosome (see figure legends for details) in 15 μl of a reaction mix of 20-mM Hepes (pH 7.9), 100-mM NaCl, 50-mM KCl, 1-mM MgCl2, 0.1-mM ethylenediaminetetraacetic acid (EDTA), 1-mM DTT, 5% (v/v) glycerol, 0.5 mg/ml of BSA and 0.1% (v/v) NP-40. Naked DNA samples were prepared in the same buffer conditions and with the same concentration of chicken erythrocyte nucleosomes used as carrier as those of nucleosomal samples. Samples were incubated at 4°C for 90 min and then run on native 4.5% polyacrylamide gels (37.5:1, 0.5 × TBE) or on a 0.8% (w/v) agarose gel (0.2 × TBE) as described (43). Gels were dried and exposed to PhosphorImager screens and quantitated using ImageQuant (Amersham Biosciences).

### Chromatin assembly and micrococcal nuclease analysis

The 601-Tel DNA fragment was obtained as described (34). The pCMV-601Telo plasmid was digested with Alw44I and the resulting 4400-bp DNA fragment was gel purified and then digested with Bsp1407I to generate a fragment of ∼2000 bp, which was gel-purified and terminally labeled by filling in with Klenow enzyme and [α-^32^P]dATP. For the assembly reaction 1 μg of labeled DNA was assembled with *Drosophila* embryo extracts essentially as described (34). To the DNA, we added 40-μl embryo extract, 40-μl EX buffer (10-mM Hepes-KOH (pH 7.6), 1.5-mM MgCl2, 80-mM KCl, 0.5-mM EGTA, 10% glycerol, 10-mM b-glycerophosphate, 1-mM DTT, 0.05% NP40), 10 μl of an energy-regenerating-system (300-mM creatine phosphate, 10-mg/ml creatine kinase, 30-mM MgCl2, 10-mM DTT, 30-mM ATP pH 8), to reach a total volume of 100 μl. After 4–6 h of assembly at 26°C, chromatin was digested for 10 min with MNase (SIGMA) at 190-U/ml concentrations. MNase reaction was stopped by adding one volume of TEES/proteinase K (10-mM Tris HCl pH 7.5, 10-mM EDTA, 10-mM EGTA, 1% SDS, 50-μg/ml proteinase K) and incubated at 37°C for 2 h to overnight. DNA was phenol-extracted and run on a 1.3% agarose gel in 1X Tris-Glycine at 1 V/cm for at least 3 h. Gels were dried and exposed to PhosphorImager screens and quantitated using ImageQuant (Amersham Biosciences). Alternatively, southern blot analysis was performed to detect unlabeled samples. Gels were submerged and shaken in 0.25-M HCl, neutralized in 1-M Tris-HCl, pH 8.0, then denatured in 1.5-M NaCl, 0.5-M NaOH and transferred to a Hybond-N membrane in 1.5-M NaCl, 0.5-M NaOH. The DNA was UV-crosslinked to the membrane, which was then pre-hybridized in Church's Buffer [0.5-M phosphate buffer, 1-mM EDTA, 7% SDS, 1% BSA] for 1 h at 60°C followed by hybridization with [α-^32^P] TTAGGG repeat or 601 probe labeled by random priming, at 60°C for at least 3 h. The membrane was washed at 60°C, in 2X SSC for 10 min, in 2X SSC/0.1% SDS for 20 min and two times in 0.1X SSC/0.1% SDS for 20 min.

## RESULTS

### TRF2 binds with low affinity to nucleosomal binding sites

**Figure 1.**
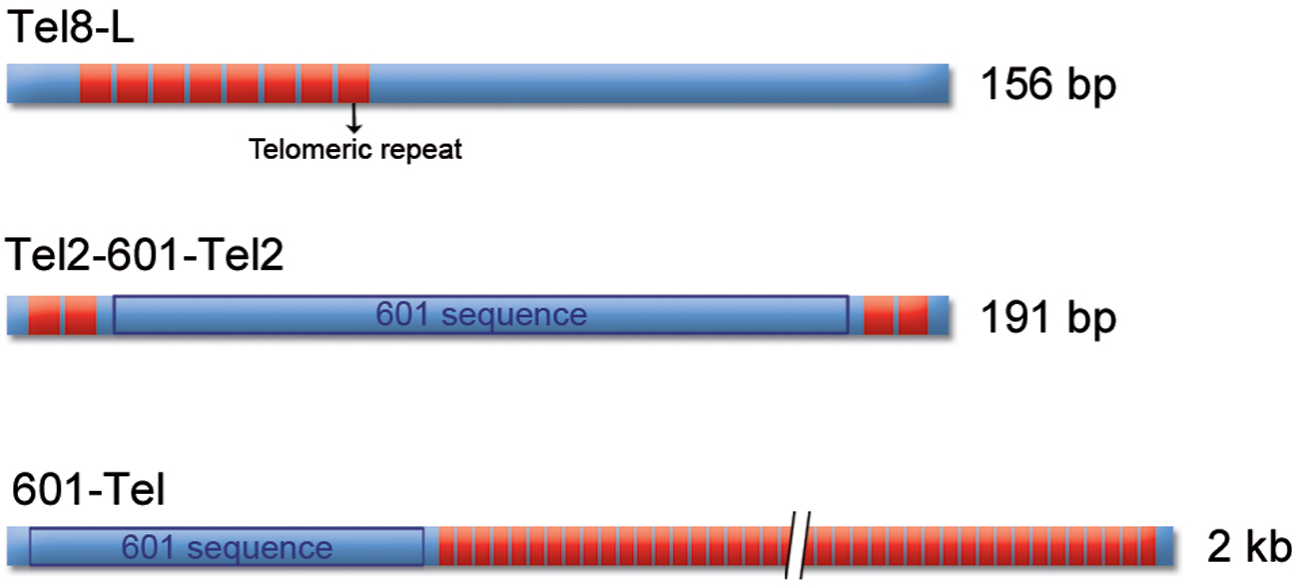
A schematic representation of the DNA fragments used in the experiments.

**Figure 2.**
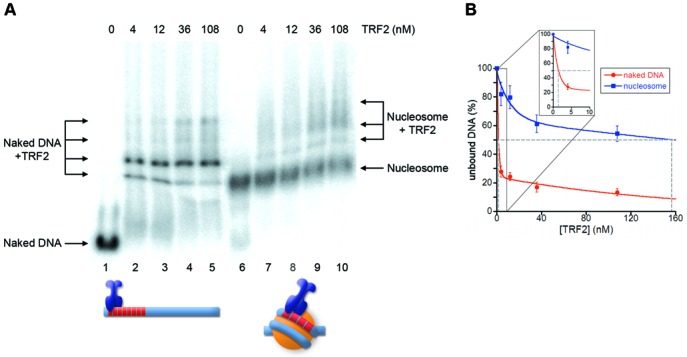
TRF2 binds to nucleosomal telomeric sequences with low affinity. (**A**) Gel mobility-shift assay. Naked DNA (lanes1–5) and NCPs formed on Tel8-L (lanes 6–10) were incubated with increasing amounts of TRF2. The molar concentration of TRF2 is indicated above the lanes. Samples were separated on a 0.8% agarose gel. (**B**) Percentages of unbound DNA and NCPs are reported as a function of protein concentration in the reaction. The concentration of TRF2 at which 50% of naked DNA and nucleosome remain unbound has been extrapolated.

In a previous work, we studied the ability of TRF1 to recognize its binding sites on the nucleosome (44). We found that TRF1 interacted with nucleosomal binding sites with a 6-fold lower affinity than naked DNA, altering the nucleosome surface structure. To test the ability of TRF2 to recognize its binding sites in a nucleosomal context, we used the same probe, Tel8-L (Figure 1), a 156-bp DNA fragment containing eight telomeric repeats located between 15 and 62 bp from one end of the fragment. Tel8-L was terminally labeled and reconstituted *in vitro* to form nucleosome core particles (NCPs) by the salt dilution method (44). The reconstituted nucleosome and the Tel8-L naked DNA fragment were incubated with increasing amounts of TRF2, and the samples run on an agarose gel to separate the various complexes. Figure 2A shows a typical electrophoretic mobility shift assay of TRF2 binding to naked DNA and NCP. Binding of TRF2 to naked DNA causes the disappearance of the naked DNA band and the simultaneous appearance of lower mobility bands that may correspond to the binding of one to four TRF2 dimers (Figure 2A, lanes 2–5). On the contrary, when TRF2 is incubated with the NCP formed on Tel8-L most of the nucleosome population remains unbound; only at high protein concentration two shifted bands appear (Figure 2A, lanes 7–10). The low affinity of TRF2 for nucleosomal binding sites makes difficult to calculate the affinity differences. Approximate values are reported in Figure 2B that reports the quantification of TRF2 binding to Tel8-L DNA and NCP from three independent experiments. The extrapolation of the protein concentration at which 50% of DNA or NCP is bound indicates that the affinity of TRF2 for nucleosomal binding sites (blue line) is reduced about 100-fold with respect to naked DNA (red line). This behavior is strikingly different from that of TRF1 (44); although the affinities of TRF1 and TRF2 for naked DNA are in the same order of magnitude, TRF1 binds on the nucleosome with only a 6-fold affinity decrease with respect to naked DNA. To rule out that the lack of binding between Tel8-L NCPs and TRF2 might depend on an inadequate nucleosomal substrate, we performed a binding experiment in parallel with TRF1 (Supplementary Figure S3). Also in this experiment is clearly visible that TRF2 does not bind nucleosomal binding sites at a protein concentration in which TRF1 shows efficient binding to NCP and in which all naked DNA is bound by TRF2.

### The DNA binding domains of TRF1 and TRF2 recognize telomeric repeats on the nucleosome with the same affinity

**Figure 3.**
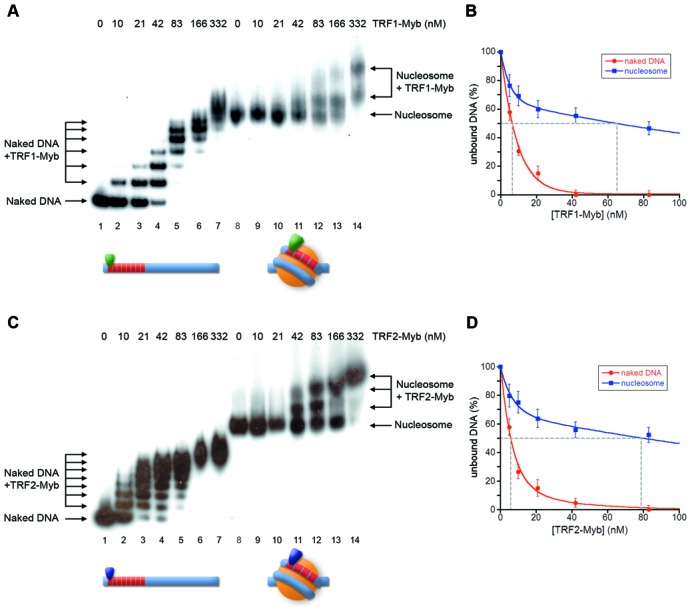
TRF1-Myb and TRF2-Myb bind to nucleosomal telomeric sequences with similar affinities. (**A, C**) Gel mobility-shift assay. Naked DNA (lanes 1–7) and NCPs formed on Tel8-L (lanes 8–14) were incubated with increasing amounts of TRF1-Myb (A) or TRF2-Myb (C). The molar concentration of TRF1-Myb and TRF2-Myb is indicated above the lanes. Samples were separated on a 4.5% polyacrylamide gel. (**B, D**) Percentages of unbound DNA and NCPs are reported as a function of protein concentration in the reaction. The concentration of TRF1-Myb (B) and TRF2-Myb (D) at which 50% of naked DNA and nucleosome remain unbound has been extrapolated.

To investigate whether the different affinity of TRF1 and TRF2 for nucleosomal binding sites could be attributed to a differential interaction of their binding domains, we performed binding assays of the Myb-SANT DNA binding domains of TRF1 and TRF2 (TRF1-Myb and TRF2-Myb) with the NCPs formed onto Tel8-L. The experiments are reported in Figure 3. We found that TRF2-Myb has a slightly higher affinity for naked DNA than TRF1-Myb; both proteins bind to the nucleosome with a lower affinity than to naked DNA. The decreased affinity can be quantified as 11-fold for TRF1-Myb and 13-fold for TRF2-Myb. These data indicate that the different binding behavior of TRF1 and TRF2 does not depend on their DNA binding domains that interact with telomeric sites on the nucleosome with similar affinities.

### TRF1 and TRF2 binding to naked DNA is affected by the presence of an adjacent nucleosome

**Figure 4.**
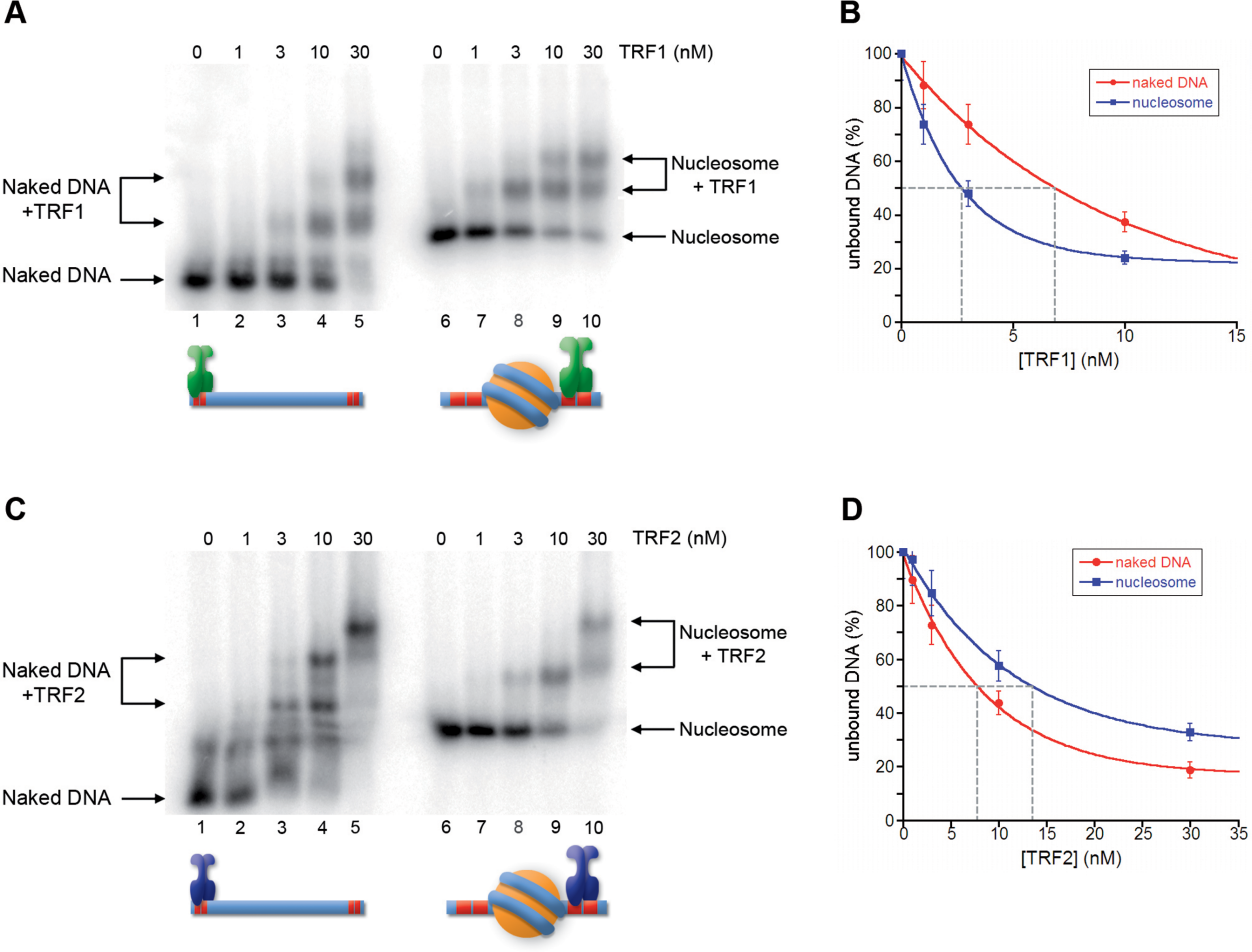
Nucleosome influences binding of TRF1 and TRF2 to adjacent DNA. (**A, C**) Gel mobility-shift assay. Naked DNA (lanes 1–5) and NCPs formed on Tel2-601-Tel2 (lanes 6–10) were incubated with increasing amounts of TRF1 (A) or TRF2 (C). The molar concentration of TRF1 and TRF2 are indicated above the lanes. Samples were separated on a 0.8% agarose gel. (**B, D**) Percentages of unbound DNA and NCPs are reported as a function of protein concentration in the reaction. The concentration of TRF1 (B) and TRF2 (D) at which 50% of naked DNA and nucleosome remain unbound has been extrapolated.

The tight nucleosomal organization found at human telomeres leaves only 10–15 bp of naked DNA between adjacent nucleosomes (32–34). This suggests that nucleosomes could represent a steric hindrance to the binding of TRF1 and TRF2 also to linker telomeric DNA. While several papers addressed the issue of whether and how proteins recognize their binding sites on the nucleosome (43,54,55), the accessibility of DNA sequences immediately adjacent to a nucleosome has been scarcely explored. To approach this question, we generated a DNA construct, Tel2-601-Tel2 (Figure 1), containing the strong positioning sequence 601 flanked by two telomeric repeats at both sides. The 601 DNA is a 147-bp synthetic sequence (56), which forms a very stable and highly positioned nucleosome (57,58). Once reconstituted, the Tel2-601-Tel2 NCP represents a model for studying the binding of TRF proteins to naked DNA in a chromatin environment. Figure 4 reports the binding of TRF1 and TRF2 to Tel2-601-Tel2 NCPs. Surprisingly, TRF1/NCP ternary complexes appear at a lower concentration of protein than TRF1/DNA complexes (Figure 4A). The quantification reported in Figure 4B indicates an affinity of TRF1 for Tel2-601-Tel2 NCP about 3-fold higher than for the corresponding naked DNA. On the contrary, in the case of TRF2 we observed a decreased affinity (∼2-fold) for TTAGGG repeats adjacent to a nucleosome formed on the 601 DNA (Figure 4C and D). Thus, the presence of a nucleosome favors TRF1 binding on adjacent naked DNA and disfavors TRF2 binding.

### Histone N-terminal tails modulate the binding of TRF1 and TRF2 in a chromatin environment

**Figure 5.**
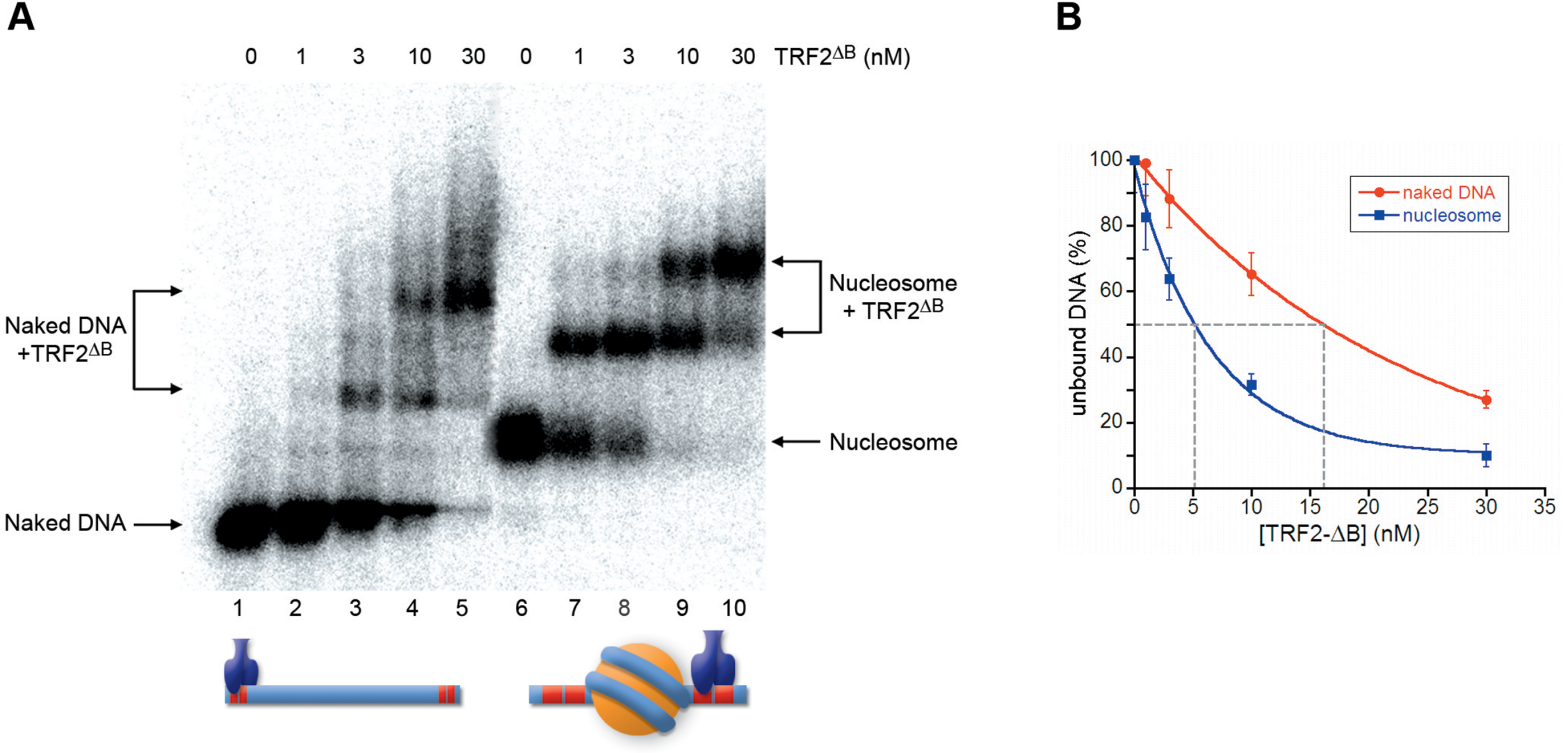
Nucleosomes favor binding of TRF2^ΔB^ to adjacent telomeric binding sites. (**A**) Gel mobility-shift assay. Naked DNA (lanes 1–5) and NCPs (lanes 6–10) were incubated with increasing amounts of TRF2^ΔB^. The molar concentration of TRF2^ΔB^ is indicated above the lanes. Samples were separated on a 0.8% agarose gel. (**B**) Percentages of unbound DNA and NCPs are reported as a function of protein concentration in the reaction. The concentration of TRF2^ΔB^ at which 50% of naked DNA and nucleosome remain unbound has been extrapolated.

**Figure 6.**
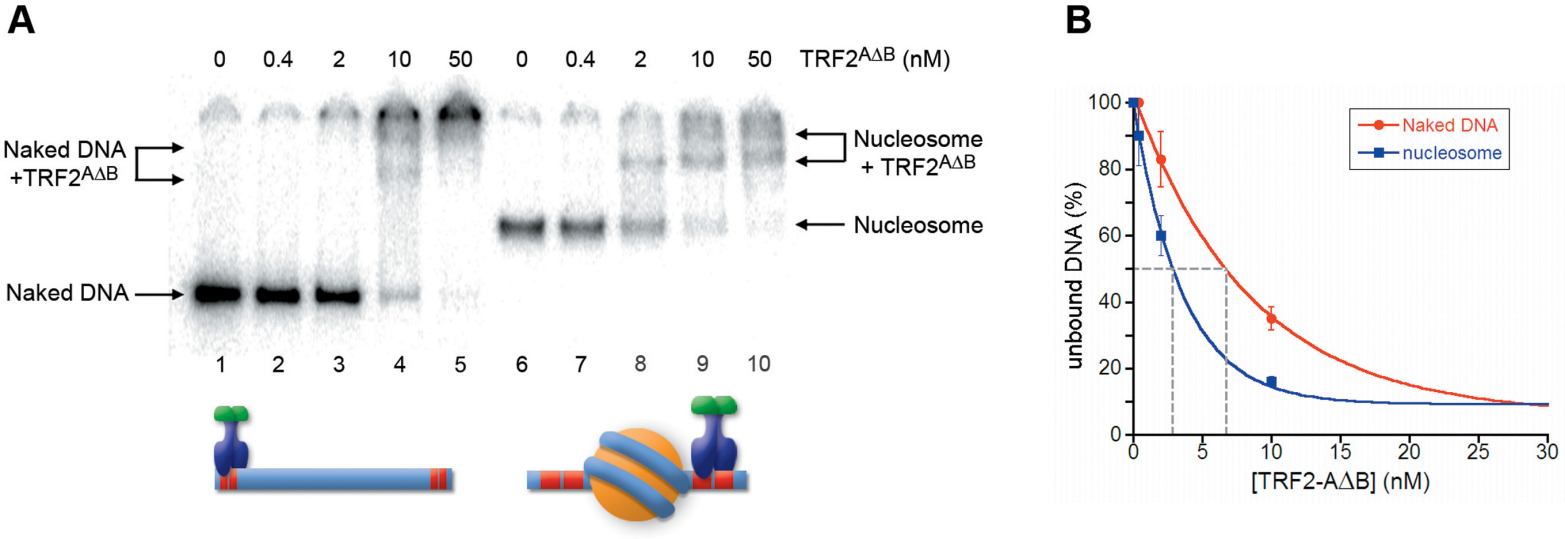
Nucleosomes favor binding of TRF2^AΔB^ to adjacent telomeric binding sites. (**A**) Gel mobility-shift assay. Naked DNA (lanes 1–5) and NCPs (lanes 6–10) were incubated with increasing amounts of TRF2^AΔB^. The molar concentration of TRF2^AΔB^ is indicated above the lanes. Samples were separated on a 4.5% polyacrylamide gel. (**B**) Percentages of unbound DNA and NCPs are reported as a function of protein concentration in the reaction. The concentration of TRF2^AΔB^ at which 50% of naked DNA and nucleosome remain unbound has been extrapolated.

Since TRF1 and TRF2 share the same overall architecture (3,11,59), we wondered which domain could be responsible for the different binding ability of the two proteins in a nucleosomal context. The N-terminal domain is the most divergent, rich of acidic residues in the case of TRF1, and of basic residues in the case of TRF2 (3). This may suggest that electrostatic interactions between the basic histone tails and the N-terminal domains of TRF1 and TRF2 could modulate their binding to telomeric chromatin. To test this hypothesis we performed binding assay experiments using a mutant of the TRF2 protein lacking the basic N-terminal domain, TRF2^ΔB^. Although this protein seems to exhibit a lower affinity for naked DNA than the full-length version, Figure 5 shows that TRF2^ΔB^ behaves like TRF1, binding with a 3-fold higher affinity to recognition sites adjacent to a nucleosome. Next, we asked what happens if we swap the basic domain of TRF2 for the acidic domain of TRF1. Therefore, we studied the binding to naked and reconstituted Tel2-601-Tel2 of the mutant protein TRF2^AΔB^, in which the N-terminal domain of TRF2 has been exchanged with the N-terminal domain of TRF1 (13). The results of this analysis, reported in Figure 6, show a 2-fold higher affinity of TRF2^AΔB^ for NCPs.

**Figure 7.**
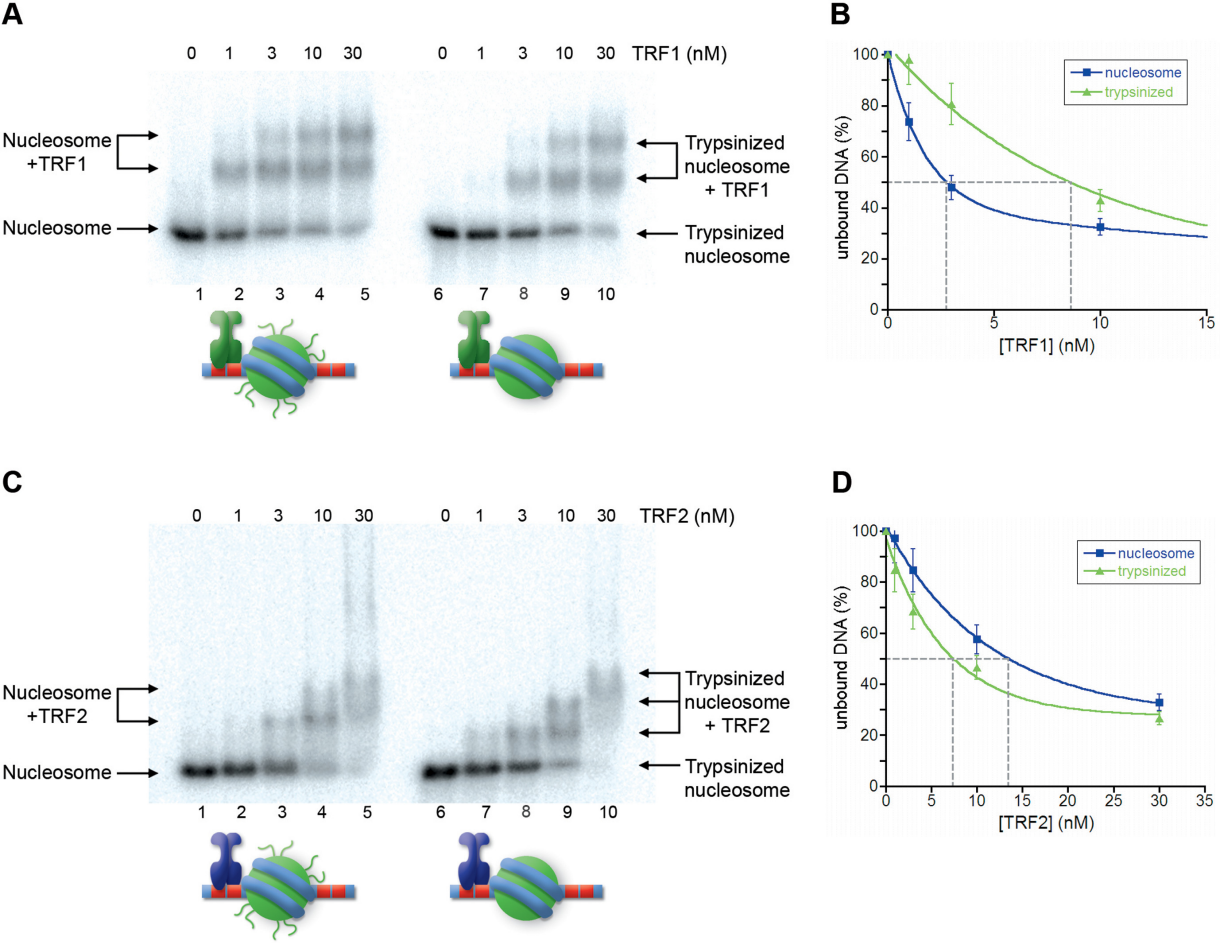
Removal of histone tails affects binding of TRF1 and TRF2 to TTAGGG repeats adjacent to NCPs. (**A, C**) Gel mobility-shift assay. Tel2-601-Tel2 NCPs (lanes1–5) and Tel2-601-Tel2 trypsinized NCPs (lanes 6–10) were incubated with increasing amounts of TRF1 (A) and TRF2 (C). The molar concentration of TRF1 and TRF2 is indicated above the lanes. Samples were separated on a 0.8% agarose gel. (**B, D**) Percentages of unbound NCPs and trypsinized NCPs are reported as a function of protein concentration in the reaction. The concentration of TRF1 (B) and TRF2 (D) at which 50% of naked DNA and nucleosome remain unbound has been extrapolated.

**Figure 8.**
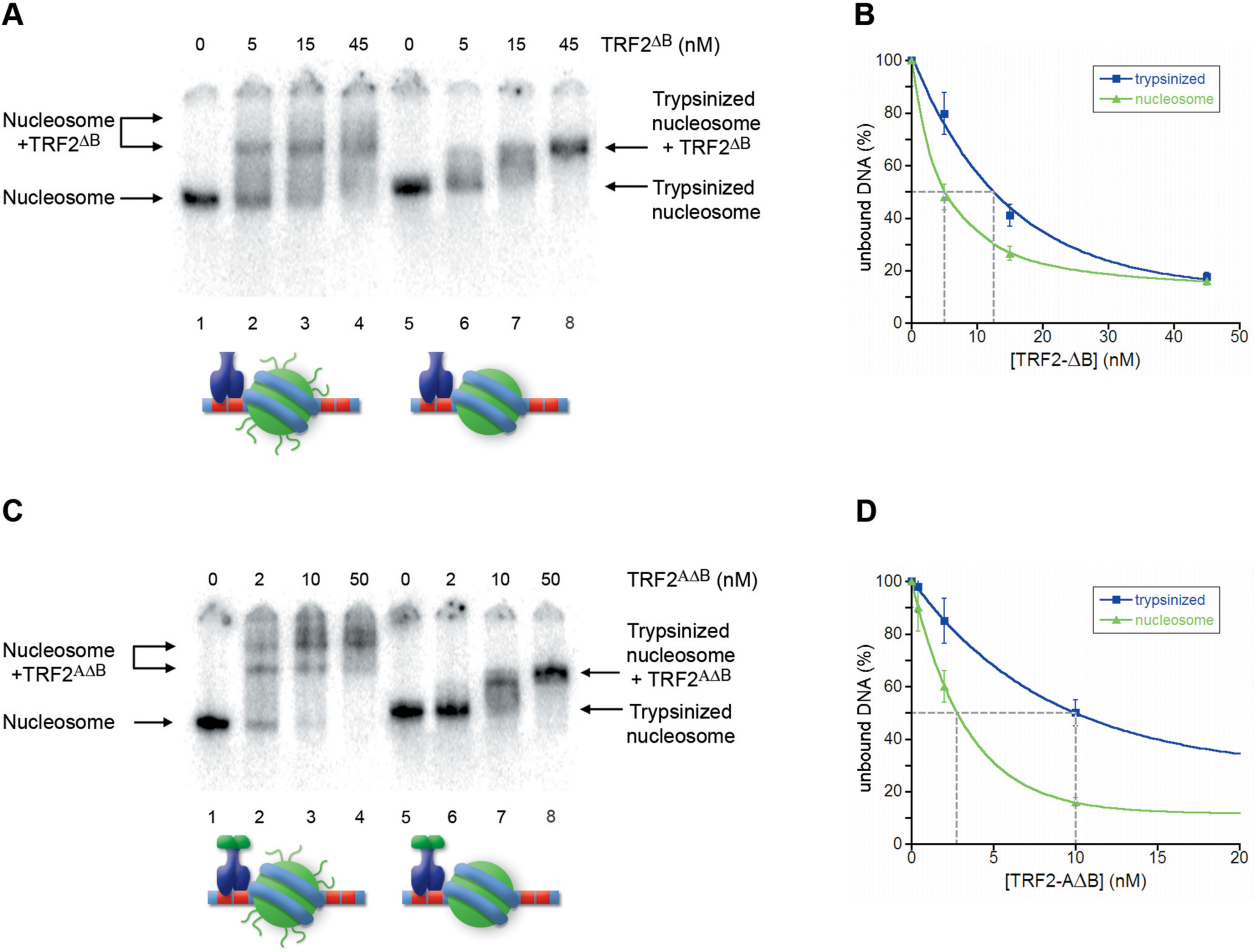
Removal of histone tails disfavors binding of TRF2^ΔB^ and TRF2^AΔB^ to TTAGGG repeats adjacent to NCPs. (**A, C**) Gel mobility-shift assay. Tel2-601-Tel2 NCPs (lanes1–5) and Tel2-601-Tel2 trypsinized NCPs (lanes 6–10) were incubated with increasing amounts of TRF2^ΔB^ (A) and TRF2^AΔB^ (C). The molar concentration of TRF2^ΔB^ and TRF2^AΔB^ is indicated above the lanes. Samples were separated on a 4.5% polyacrylamide gel. (**B, D**) Percentages of unbound NCPs and trypsinized NCPs are reported as a function of protein concentration in the reaction. The concentration of TRF2^ΔB^ (B) and TRF2^AΔB^ (D) at which 50% of naked DNA and nucleosome remain unbound has been extrapolated.

To explore the role played by histone tails and to gain further support to our hypothesis, we carried out binding assays using nucleosomes previously digested with trypsin to remove histone tails. Indeed, the quantifications show that the removal of histone tails causes a 3-fold decrease of TRF1 affinity for Tel2-601-Tel2 NCPs (Figure 7A and B), and ∼2-fold increase in the case of TRF2 (Figure 7C and D). Then, we asked whether the removal of histone tails affects the affinities of the mutants TRF2 proteins for Tel2-601-Tel2 NCPs. These experiments are reported in Figure 8. Contrarily to the agarose gels shown in Figure 7, tailless nucleosomes migrate slower than the intact NCP on polyacrylamide gels (see Supplementary Figure S4; (60)). TRF2^ΔB^ and TRF2^AΔB^ show, respectively, a 2-fold and a 3-fold diminished affinity for Tel2-601-Tel2 NCPs depleted of histone tails. Collectively, these data are consistent with a modulating role of the histone tails and of the N-terminal domains of TRF proteins in their binding to telomeric chromatin.

### TRF1 alters telomeric nucleosome spacing *in vitro*

We previously found that TRF proteins can alter telomeric nucleosome positioning and spacing. TRF1 is able to induce nucleosome sliding onto adjacent DNA in an *in vitro* model (45), whereas TRF2 is able to increase nucleosome spacing at telomeres in a concentration-dependent way (34). To investigate whether TRF1 might alter also the regular telomeric nucleosome spacing, we used an assembly system previously set up to analyze TRF2 effect on nucleosome spacing and positioning, based on *Drosophila* embryonic extracts (61) and a construct containing the 601 DNA placed upstream of a 1700-bp human telomeric DNA (601-Tel; Figure 1). Using this method, nucleosome assembly occurs in the presence of histone chaperones and ATP-dependent remodeling complexes at physiological ionic strengths, allowing the production of regularly spaced nucleosomal arrays (62). Moreover, this assembly system allows evaluating the impact of human telomeric protein on nucleosome assembly, since shelterin proteins are absent in *Drosophila*. In our previous work we reproduced the short nucleosome spacing found *in vivo* at telomeres, demonstrating that this is a sequence-specific property of telomeric sequences (34). Furthermore, we demonstrated that adding TRF2 to the assembly resulted in the increase of nucleosome spacing.

**Figure 9.**
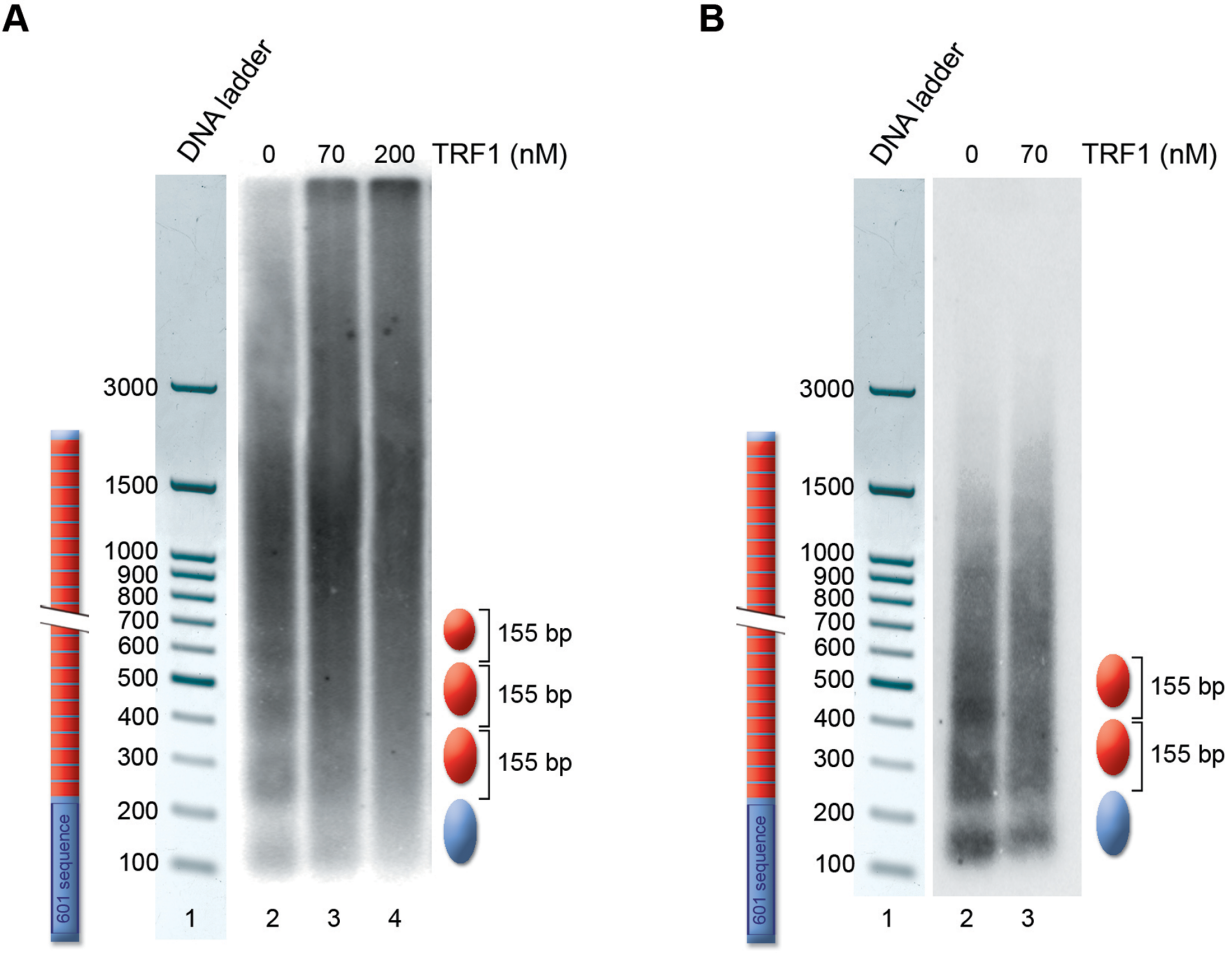
TRF1 alters telomeric nucleosome spacing *in vitro*. MNase digestion of chromatin assembled on the 601-Tel DNA fragment. (**A**) Lane 1, 100-bp DNA ladder; lane 2, chromatin assembled in the absence of TRF1; lane 3, chromatin assembled in the presence of 70-nM TRF1; lane 4, chromatin assembled in the presence of 200-nM TRF1; (**B**) Lane 1, 100-bp DNA ladder; lane 2, chromatin assembled in the absence of TRF1; lane 3, chromatin assembled in the presence of 70-nM TRF1. All samples are digested with 400 U/ml of MNase. A schematic drawing of the nucleosomal positioning and spacing is represented on the right.

Nucleosomal arrays were assembled on 601-Tel by incubating with *Drosophila* embryonic extracts both in the presence and in the absence of TRF1. Samples were digested with micrococcal nuclease (MNase) and the resulting DNA fragments separated on an agarose gel (Figure 9). Even if it is difficult to distinguish nucleosomal bands beyond the tetranucleosome because of the multiple positioning of telomeric nucleosomes (34,37,40), digestion of the telomeric nucleosomal arrays with MNase produces a 155-bp nucleosomal ladder (Figure 9A, lane 2), consistent with the spacing found *in vivo* and in previous *in vitro* experiments (32–34). The addition of TRF1 to the assembly reaction results in the alteration of the nucleosome spacing. At a protein concentration of 70 nM only the mononucleosome and the dinucleosome are detectable (Figure 9A, lane 3), whereas the remaining digestion product is smeared; at increasing TRF1 concentration the regular nucleosome spacing completely disappears (Figure 9A, lane 4). The alteration of the nucleosomal pattern remains also evident at increasing MNase digestion, as shown in Figure 9B. To rule out that the observed pattern might depend on protection by MNase digestion due to TRF1 binding, we added TRF1 after nucleosomal assembly. In this case, the nucleosomal pattern is not altered (Supplementary Figure S5A, lane 4). Furthermore, MNase digestion of TRF1 bound to naked 601-Tel DNA shows that the protein does not protect from MNase cutting (Supplementary Figure S5B). On the other hand, incubation of TRF1 with 601-Tel before adding the *Drosophila* extracts does not prevent the formation of a nucleosomal array, even if with a disordered nucleosomal spacing (Supplementary Figure S5C, lane 2). All these data suggest that TRF1 interferes with nucleosome spacing during chromatin assembly.

Then, to verify whether the remodeling effect is simply a consequence of competition for binding sites between TRF proteins and the histone octamer, we assembled nucleosomal arrays on 601-Tel in the presence of the Myb-SANT binding domains of TRF1 and TRF2 (Supplementary Figure S6). In both cases, the MNase digestion pattern is unaltered, suggesting that the intact proteins are necessary for a remodeling effect on nucleosome spacing.

## DISCUSSION

The binding of TRF1 and TRF2 to telomeres is essential to guarantee protection from DDR and DNA repair enzymes. It has been often overlooked that this happens in a tight chromatin context. It is still unknown whether all the telomeric region is organized in tightly spaced nucleosomes or otherwise part of the telomere is nucleosome-free. An analysis of the chromatin organization of mouse telomeres reports that nucleosomes are present till the very end of the telomere and that this organization is maintained in deprotected telomeres after shelterin removal (21,63). This suggests that TRF proteins have to bind on the nucleosome or on the short linker DNA between nucleosomes, competing or interacting with the histone octamer.

In this study, we addressed the matter of whether and how nucleosomes affect TRF1 and TRF2 binding to TTAGGG repeats by using different *in vitro* model systems. Besides studying the affinity of TRF proteins for nucleosomal binding sites, we investigated also whether nucleosomes influence the binding to the immediately adjacent naked DNA, that is linker DNA. To our knowledge, this issue has been approached in the case of the linker histone H1 and of other chromatin binding proteins (57,64–67), but never for a sequence-specific binding protein. Overall, we found that TRF1 and TRF2 show a different affinity for their binding sites in a chromatin environment.

### TRF2 has a much lower affinity for nucleosomal telomeric binding sites than TRF1

Nucleosomal organization greatly reduces the accessibility of proteins to their binding sites. We previously showed that TRF1 is able to recognize its binding sites on the nucleosome with a 6-fold lower affinity with respect to naked DNA and that the formation of this ternary complex is strongly dependent on the orientation of the binding sites on the nucleosome surface (44). In contrast, TRF2 binds very poorly on the nucleosome (Figure 2), forming ternary complexes only at high protein concentration. This was somewhat unexpected, since TRF1 and TRF2 share the same architecture binding DNA as homodimers. Moreover, their binding domains recognize telomeric repeats on the nucleosome with a very similar affinity (Figure 3). It is worth noting that telomeric binding sites exposed on the nucleosome surface are non-consecutive, separated by at least one DNA helical repeat (44). Therefore, binding to nucleosomal DNA of the TRF1 dimer might be facilitated by its high spatial flexibility that allows the two binding domains of the homodimer to interact independently with non-consecutive telomeric repeats (20).

### Nucleosome affects TRF1 and TRF2 binding on adjacent linker DNA

Given that telomeric nucleosomes are connected by a very short 10–15-bp long linker DNA, we wondered whether TRF1 and TRF2 binding to naked DNA could be hindered by the presence of adjacent nucleosomes. We addressed this issue by generating a model for the binding to linker DNA consisting of a nucleosome formed on the strong positioning sequence 601 flanked by two telomeric repeats at each side. Our experiments showed that TRF2 affinity for naked DNA decreases by a factor of two in the presence of an adjacent nucleosome, while TRF1 affinity increases (Figure 4). This different behavior might be attributed to electrostatic interactions between the basic histone tails and the N-terminal tail of TRF1 (rich of acidic residues) and of TRF2 (rich of basic residues). The experiments shown in Figures 5–8 support this hypothesis: upon removal of histone N-terminal tails the affinities of TRF1 and TRF2 for Tel2-601-Tel2 NCPs resemble those of naked DNA. Further support comes from the interaction with Tel2-601-Tel2 NCPs of a mutant TRF2 protein lacking the N-terminal basic domain, TRF2^ΔB^ and of the protein TRF2^AΔB^, in which the acidic domain of TRF1 replaces the basic N-terminal domain of TRF2. In both cases the affinity for telomeric sites is higher in the case of NCP than for naked DNA (Figures 5 and 6). However, the 3-fold higher affinity of TRF2^ΔB^ for sites close to a nucleosome with respect to naked DNA (Figure 5) cannot be explained only with repulsive interactions between histone tails and the basic N-terminal domain of TRF2. In addition, the affinity of TRF2^ΔB^ for Tel2-601-Tel2 NCPs shows a 2-fold decrease after removal of histone N-tails (Figure 8A). These data suggest that other interactions are involved. It is possible that other domains of the protein—the dimerization domain and/or the hinge domain—could interact with nucleosomes with a positive effect on TRF2 DNA binding affinity. In addition, since TRF2 is known to bind preferentially unusual DNA structures such as supercoiled DNA and Holliday junctions (14,16), it might recognize with a different affinity the distorted structure of the DNA linker at the entry–exit of the nucleosome.

### TRF1 disrupts the spacing of telomeric nucleosomes

Previous works showed that TRF2 overexpression in mouse (46) and human cells (34), and addition of purified TRF2 protein *in vitro* to an assembly system based on *Drosophila* embryonic extracts (34), results in the increase of nucleosome spacing at telomeres. This is consistent with a dynamical view of telomeric chromatin and with the intrinsic sequence-dependent mobility of telomeric nucleosomes (41). The experiment shown in Figure 9 indicates that TRF1 is also able to alter nucleosome spacing during nucleosomal assembly in the presence of histone chaperones and remodeling enzymes. The alteration of nucleosome spacing by TRF1 and TRF2 could simply derive from the competition with the histone octamer for binding to TTAGGG repeats. However, when TRF1-Myb and TRF2-Myb are included in the assembly the nucleosome spacing is unaltered (Supplementary Figure S8), indicating that the remodeling activities are properties of the entire TRF proteins. Moreover, it is worth noting that whereas the main effect of TRF2 addition to the assembly system is the increase of nucleosome spacing (34), TRF1 seems to completely deregulate the organization of the telomeric nucleosomal array. The higher remodeling activity of TRF1 might reflect the different way TRF1 and TRF2 interact with chromatin. In nucleosome assembly, regular spacing is assured by the activity of ATP-dependent chromatin remodeling enzymes (68); the combination of the nucleosome intrinsic propensity to slide on telomeric sequences (41) and the ability of TRF1 to further induce *in vitro* this sliding (45) might generate completely deregulated nucleosome spacing. In our *in vitro* model, TRF1 is not able to interfere with nucleosome spacing if added after the nucleosomal array is formed, suggesting that it might affect chromatin organization mainly at the level of nucleosome assembly. In a previous work we showed that TRF2 induces the reorganization of telomeric chromatin in a cell cycle regulated event occurring at the end of the S phase (34). It is reasonable to suppose that remodeling of telomeric chromatin organization might be connected with DNA replication, when nucleosomes are disrupted ahead of the replication fork and reassembled behind. TRF proteins are constantly present at telomeres during all the phases of the cell cycle, even if in primary human fibroblasts the level of TRF1 bound at telomeres decreases during S-phase and G2, while TRF2 slightly decreases during G2 (69). When telomeres shorten, the competition between nucleosomes and TRF proteins for the reduced number of telomeric sequences increases. In this context, the ability of both TRF1 and TRF2 to compete with nucleosomes and alter telomeric chromatin organization as a function of their concentration might have an important role in the structural changes linked to telomere shortening. During nucleosome assembly, this could result in an increased spacing between nucleosomes.

## CONCLUSIONS

The cell fate upon telomere shortening is finely regulated by the equilibrium between protected versus deprotected state and depends mainly on shelterin binding (particularly TRF2) at chromosome ends. Nucleosomes hinder binding on nucleosomal telomeric sequences—particularly in the case of TRF2—and influence the interaction on adjacent linker DNA, favoring the binding of TRF1 and disfavoring TRF2 (Table 1). From our data it emerges that TRF1 alters nucleosome assembly in a concentration-dependent way; moreover, it binds preferentially on the linker DNA between nucleosomes, but is also able to recognize nucleosomal binding sites. Instead, TRF2 has a very low affinity for binding sites on the nucleosomes and prefers binding naked DNA in nucleosome-free regions. Importantly, we showed here that TRF1 and TRF2 binding are modulated by nucleosomes via the histone tails. This suggests that changes in the chromatin structure of telomeres might influence the accessibility of TRF proteins to their binding sites by altering the charge environment. Recently, a chromatin immunoprecipitation-exonuclease (ChIP-exo) study in yeast showed that histone H3 tails interact with linker DNA (70); these interactions are negatively regulated by H3K36me3. These findings suggest that the binding of specific proteins to their sites on linker DNA is affected by nucleosomes and post-translational histone modifications. Therefore, changes of the histone marks pattern at telomeres might influence the binding of shelterin proteins and telomeric chromatin compaction.

**Table 1.** *C*_1/2_ values for DNA and nucleosomal substrates^a^

	Tel8-L		Tel2-601-Tel2		
Proteins	Naked DNA	NCP	Naked DNA	NCP	NCP_Tryp_
TRF1	0.9 ± 0.2^b^	5.8 ± 0.2^b^	7.0 ± 1	2.5 ± 0.5	8.2 ± 1
TRF2	1.5 ± 0.5	160 ± 30	7.6 ± 0.5	13 ± 2	7.5 ± 1
TRF1-Myb	6.0 ± 1	65 ± 10			
TRF2-Myb	6.1 ± 1	77 ± 10			
TRF2^ΔB^			16 ± 3	5.1 ± 1	12 ± 2
TRF2^AΔB^			7.0 ± 1	3.0 ± 0.5	10 ± 1

^a^The *C*_1/2_ value represents the protein concentration at which 50% of the substrate is bound.

^b^Values reported in ref. (44).

Although a clear picture of the chromatin structure of human telomeres is still lacking, recent data suggest that histone hypoacetylation is essential for a protected telomeric state (48). In mice, telomere shortening is accompanied by a decrease of heterochromatic marks and in the increase of histone acetylation (47). Important modifications of histone tails are consequent to the establishment of a DDR signaling, namely phosphorylation of Ser139 and ubiquitination of H2A and H2AX. It might be hypothesized that changes in histone marks at telomeres might not only represent a signal for regulatory proteins but also alter the way TRF proteins interact with telomeric chromatin. Acetylation of lysine residues reduces the overall positive charge of the histone tails, destabilizes higher-order chromatin structures (71–73) and increases the accessibility of nucleosomal DNA to binding proteins (38,74,75). The charge of histone tails is also altered by phosphorylation of serines and threonines by introducing negatively charged phosphate groups. If the different affinity of TRF1 and TRF2 in a nucleosomal context depends on electrostatic interactions between TRF N-terminal domains and histone tails, then both acetylation and phosphorylation might favor TRF2 and disfavor TRF1 binding. On the other site, modifications such as ubiquitination and sumoylation introduce bulky groups on lysine residues that might increment the steric hindrance of nucleosomes.

Our results show that binding of TRF proteins—and therefore shelterin—is affected by the telomeric chromatin context. We suggest that chromatin epigenetic changes and the relative concentration of TRF proteins and the histone octamer might exert a role in regulating shelterin binding to telomeres and therefore in the steps leading from a protected to a deprotected telomeric state.

## SUPPLEMENTARY DATA

Supplementary Data are available at NAR Online.

SUPPLEMENTARY DATA
