# Supplementary material for: TRF1 and TRF2 binding to telomeres is modulated by nucleosomal organization
Source: Nucleic Acids Res. 2015 May 20;43(12):5824–37. doi: 10.1093/nar/gkv507 (PMC4499135; doi:10.1093/nar/gkv507)
Supplement: SUPPLEMENTARY DATA [file supp_43_12_5824__index.html]

TRF1 and TRF2 binding to telomeres is modulated by nucleosomal organization — TRF1 and TRF2 binding to telomeres is modulated by nucleosomal organization — SUPPLEMENTARY DATA 

# TRF1 and TRF2 binding to telomeres is modulated by nucleosomal organization

## SUPPLEMENTARY DATA

- SUPPLEMENTARY DATA
