# Supplementary material for: TRF1 and TRF2 binding to telomeres is modulated by nucleosomal organization
Source: Nucleic Acids Res. 2015 May 20;43(12):5824–37. doi: 10.1093/nar/gkv507 (PMC4499135; doi:10.1093/nar/gkv507)
Supplement: SUPPLEMENTARY DATA [file supp_gkv507_nar-02447-v-2014-File011.pdf]

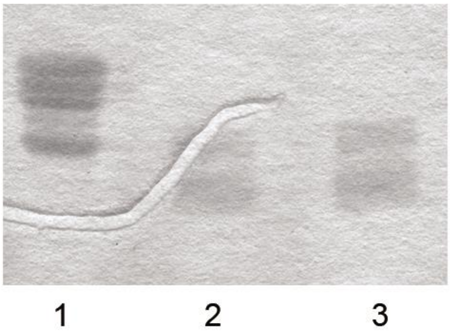

Figure S1. Characterization of normal and tailless nucleosomes. Coomassie-stained 18% SDS−PAGE showing core histones before (lane 1) and after (lanes 2 and 3) digestion with trypsin.

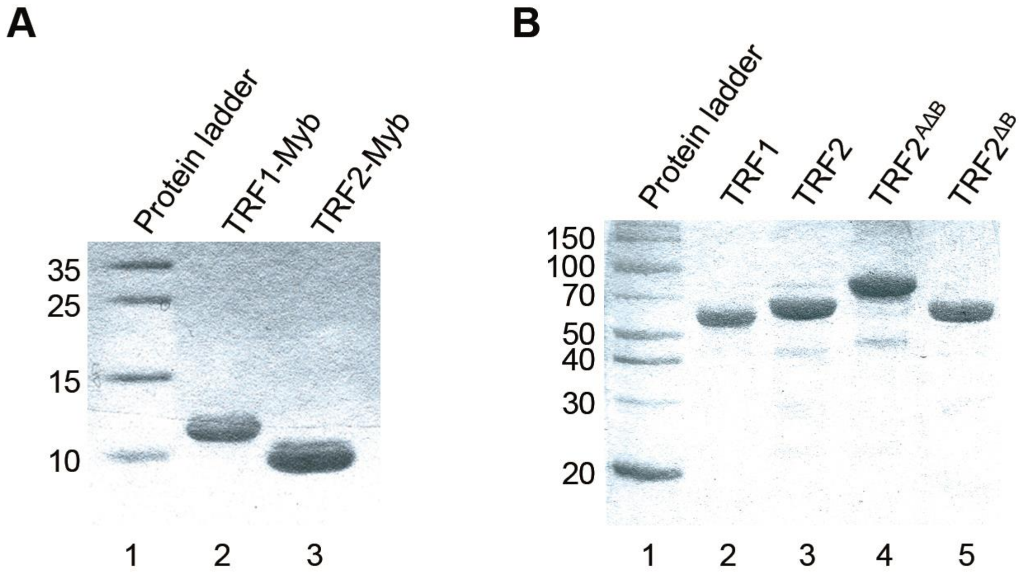

Figure S2. SDS-PAGE gel analysis of the recombinant proteins after purification. (A) Lane1, protein ladder; lane 2, TRF1-Myb; lane 3, TRF2-Myb. (B) Lane1, protein ladder; lane 2, TRF1; lane 3, TRF2; lane 4, TRF2$^{A\Delta B}$; lane 5, TRF2$^{\Delta B}$.

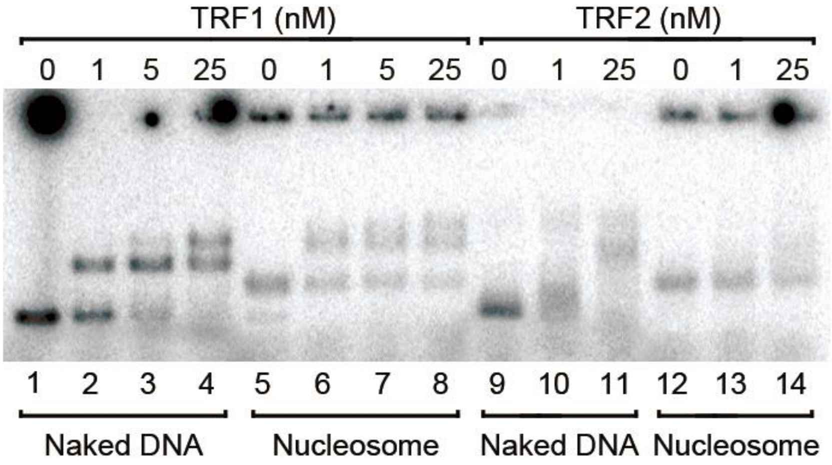

Figure S3. Binding of TRF1 and TRF2 to Tel8-L NCPs. Gel mobility-shift assay. Naked DNA (lanes 1–4 (TRF1) and 9-11 (TRF2)) and NCPs formed on Tel8-L (lanes 5–8 (TRF1) and 12-14 (TRF2)) were incubated with increasing amounts of TRF1 and TRF2. The molar concentration of TRF1 and TRF2 is indicated above the lanes. Samples were separated on a 0.8% agarose gel.

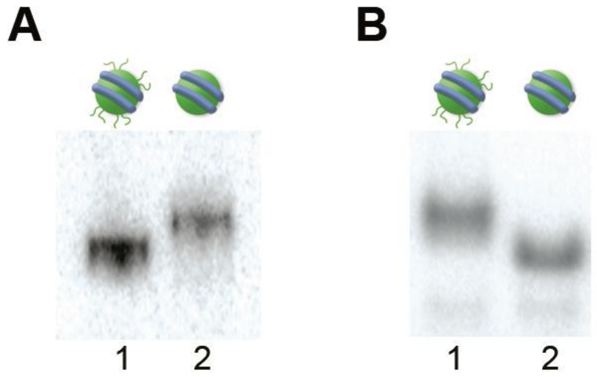

Figure S4. Electrophoretic migration of normal and tailless nucleosomes. Lane 1, normal nucleosome; lane 2, tailless nucleosome. (A) Migration on polyacrylamide gel. (B) Migration on agarose gel.

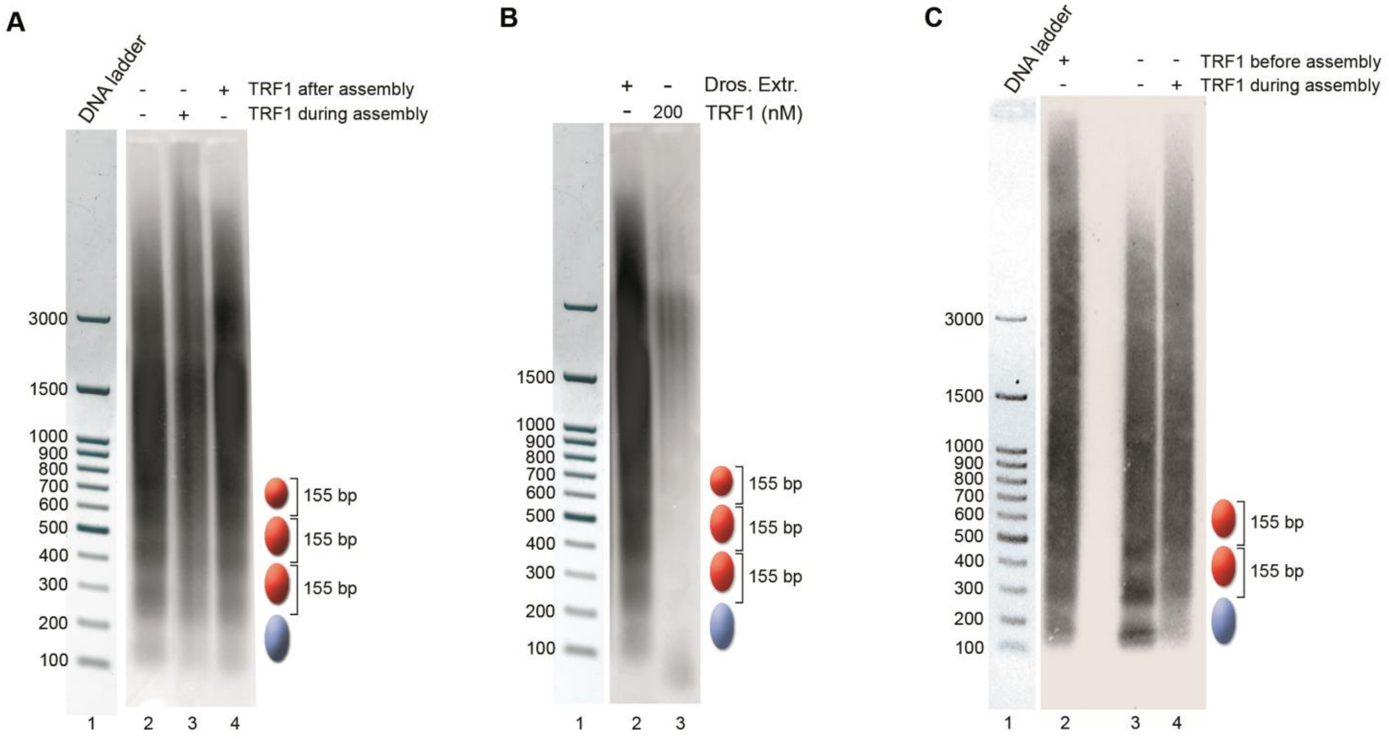

Figure S5. TRF1 alters nucleosome spacing during chromatin assembly. See text for details. All samples are digested with 190 U/ml of MNase. A schematic drawing of the nucleosomal positioning and spacing is represented on the right. (A) Lane 1, 100 bp DNA ladder; lane 2, chromatin assembled in the absence of TRF1; lane 3, chromatin assembled in the presence of 100 nM TRF1; lane 4, chromatin first assembled and then incubated for one hour with 100 nM TRF1. **(B)** Lane 1, 100 bp DNA ladder; lane 2, chromatin assembled in the absence of TRF1; lane 3, 601-Tel DNA fragment incubated with 200 nM TRF1. **(C)** Lane 1, 100 bp DNA ladder; lane 2, 601-Tel DNA fragment incubated for one hour with 100 nM TRF1, then assembled by adding the Drosophila extract; lane 3, chromatin assembled in the absence of TRF1; lane 4, chromatin assembled in the presence of TRF1.

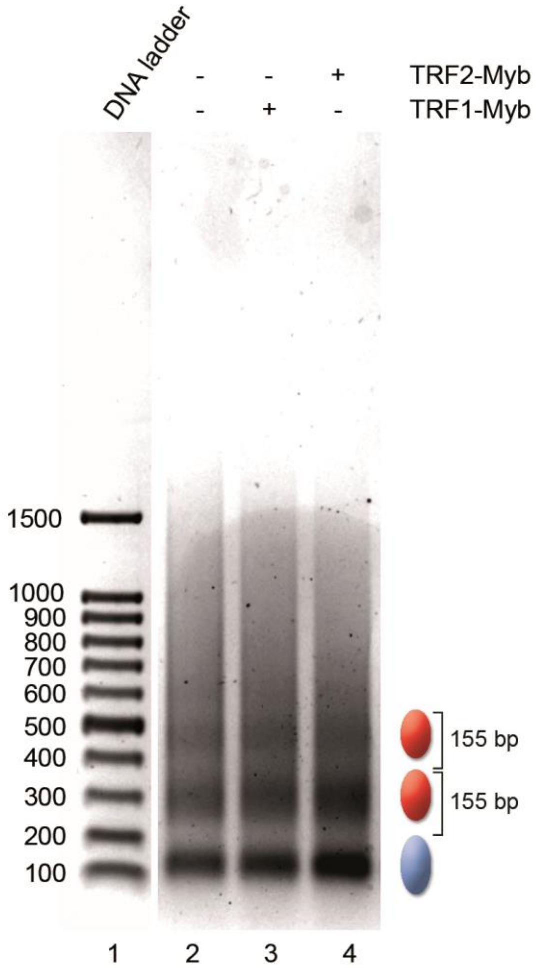

Figure S6. TRF1-Myb and TRF2-Myb do not alter telomeric nucleosomal spacing. Lane 1, 100 bp DNA ladder; lane 2, chromatin assembled on 601-tel DNA fragment; lane 3, chromatin assembled in the presence of 100 nM TRF1-Myb; lane 4, chromatin first assembled in the presence of 100 nM TRF2-Myb. All samples are digested with 190 U/ml of MNase. A schematic drawing of the nucleosomal positioning and spacing is represented on the right.
